# Burnout among young Italian Internists: a cross-sectional study assessing prevalence and associated factors

**DOI:** 10.1007/s11739-025-03978-4

**Published:** 2025-05-25

**Authors:** Gabriele Angelo Vassallo, Tommaso Dionisi, Monica Maria Failla, Adwoa Agyei-Nkansah, Davide Carrara, Filomena Pietrantonio, Caterina Delcea, Miguel Romano, Giovanni Talerico, Rosanna Villani, Giulio Francesco Romiti, Giulia Crisci, Giuseppe Augello, Alberto Maria Marra, Giovanni Addolorato, Andrea Salzano

**Affiliations:** 1 Internal Medicine Unit, Barone Lombardo Hospital, Canicattì, AG Italy; 2 Department of Medical and Surgical Sciences, Università Cattolica Di Roma, Rome, Italy; 3 Department of Internal Medicine, University of Ghana, Accra, Ghana; 4 Department of Internal Medicine, Versilia Hospital, Lido Di Camaiore, Italy; 5 Internal Medicine Unit, Medical Area Department, Castelli Hospital, ASL Roma 6, Rome, Italy; 6 Cardiology Department, Carol Davila, University of Medicine and Pharmacy, Bucharest, Romania; 7 Intensive Care Unit, Unidade Local de Saúde Do Alto Minho (ULSAM), School of Medicine, Minho University, Braga, Portugal; 8 Medicina Interna, Azienda Ospedaliera San Giovanni Addolorata, Rome, Italy; 9 Liver Unit, Department of Medical and Surgical Sciences, University of Foggia, Foggia, Italy; 10 Department of Translational and Precision Medicine, Sapienza-University of Rome, Rome, Italy; 11 Division of Cardiology, University of Milano School of Medicine, San Paolo Hospital, Milan, Italy; 12 Department of Translational Medical Sciences, Federico II University, Naples, Italy; 13 Internal Medicine and Alcohol Related Disease Unit, Columbus-Gemelli Hospital, Fondazione Policlinico Universitario A. Gemelli IRCCS, Rome, Italy; 14 Interdepartmental Center for Gender Medicine Research “GENESIS”, Federico II University, Naples, Italy; 15 Cardiology Unit, AORN A Cardarelli, Naples, Italy

**Keywords:** Burnout, Young internists, Internal medicine residents, Soft skills, Support system

## Abstract

Burnout among physicians is associated with poor productivity at work, dissatisfaction, and regrets about career choices. The present study aims to evaluate the prevalence of burnout and its main associated factors among young Italian Internists. An online survey assessing different aspects of training and working conditions of young internists was conducted across European countries by the Young Internists Group of the European Federation of Internal Medicine (EFIM). Only data from Italian respondents were analyzed in the present study. Data from 106 young Internists (aged < 40 years) working in university and non-teaching hospitals were abstracted. The median age was 30 years. Burnout was reported by 37.2% of the respondents. The main associated factors with burnout were being an Internal Medicine Resident, working in a university hospital, working extra hours than required by the hospital, managing both inpatients and outpatients, stress from inadequate time for social activities, and high workload. The absence of training or guidance on soft skills was also associated with burnout. Only 2.8% of the respondents reported the presence of a support system to deal with burnout in their hospitals. Burnout is very common among young Italian Internists with few support systems incorporated into the hospital set up. Holistic strategies to prevent or reduce burnout including modifying associated factors, should be implemented. Training for transversal skills should be added to the curriculum of internal medicine residents. Finally, a support system to deal with burnout should be present in every hospital.

## Introduction

The term ‘burnout’ evolved in health settings in the late ‘60 s to describe the condition of emotional and psychological stress experienced by clinicians during their work with fragile patients [1]. In the ‘80 s, Maslach and colleagues defined burnout as a combination of emotional exhaustion, depersonalization, and low personal accomplishment induced by chronic conditions of stress in physicians [2]. According to the last edition of the International Classification of Diseases (ICD-11), burnout, is defined as a syndrome caused by chronic workplace stress that has not been successfully managed [3]. It is characterized, according to the World Health Organization, by feelings of exhaustion or energy depletion, feelings of negativism or mental distance from one’s job, and finally by reduced professional efficiency [3].

The etiopathogenesis of burnout is heterogeneous, involving both internal and external factors. Internal factors include perfectionism, repression of personal needs to accomplish others’ expectations, and lack of a satisfactory social life. External factors, on the other hand, include working for a long time, increasing responsibilities, a bad work environment, and poor collaboration among colleagues [4]. Burnout develops in the context of an imbalance between the effort to complete the tasks and the reward for the work performance. The main consequences of burnout in physicians are depression, dissatisfaction with the job chosen, suboptimal quality of care provided to patients, and medical errors [5].

With the ageing of the population, especially in the Western World, Internal Medicine specialists have focused, in the last decades, on the management of more complex diseases and more fragile patients, with a consequent increase in the onset of burnout [6]. The COVID-19 outbreak has also contributed to a rise in the prevalence of burnout among Internists. Nonetheless, physicians who are board-certified in internal medicine showed a lower risk of developing emotional exhaustion compared to other members of the COVID-19 team with different specializations [7].

Among Internal Medicine Specialists, those who manage outpatient clinics only are less prone to burnout than their peers who work in hospitals. This may be a result of a comparatively better work-life balance [8]. A recent study showed a high prevalence of burnout levels among US Internal Medicine Trainees. The main risk factors for burnout included documentation time pressure and lack of work control. Moreover, female clinicians had higher odds of burnout than male clinicians [9].

There is no published data currently available looking at the prevalence of burnout in the population of young Italian Internists. This study aims to assess the prevalence of burnout in the population of young Italian internists (defined as internal medicine residents or physicians already specialized in internal medicine aged less than 40 years) and to evaluate associated factors with its onset.

## Methods

From October 2022 to March 2023 a survey, assessing different aspects of training and working conditions of young internists, was conducted across European countries by the Young Internists Group of the European Federation of Internal Medicine (EFIM). Data were collected through an online questionnaire (https://www.surveymonkey.com/r/VTXRHD6). The questionnaire was shared electronically through social media and by e-mail to young internists, belonging to the European Federation of Internal Medicine (which include Italian internists). The questionnaire, based on 60 multiple choice questions, assessed demographic characteristics, work information, opinions about challenges in internal medicine settings, hours required and effectively spent at work, number of patients managed, number of night shifts per month, presence or less of a day off after a nightshift, teaching program, soft skills, burnout, presence of a support system to face burnout in the hospital and future career choices. For the aim of the present study, only data coming from Italian respondents were analyzed. Burnout was defined as the co-presence of emotional exhaustion, depersonalization, and personal accomplishment, assessed through factorial analysis. Emotional exhaustion was captured by items about exhaustion and stress frequency, and overall workload, reflecting fatigue and stress. Depersonalization measured emotional detachment using the disconnection frequency item, while lack of personal accomplishment assessed reduced satisfaction and value through items about work fulfillment, enthusiasm frequency, and perceived work value. Factor analysis identified three latent dimensions—workload stress, professional disconnection, and personal accomplishment—confirming burnout as a composite syndrome with interrelated components.

### Statistical analysis

All analyses were conducted using Stata 18. Descriptive statistics were used to summarize respondent demographics, professional characteristics, workload factors, and burnout metrics. Categorical variables were presented as frequencies and percentages, while continuous variables were summarized using means and standard deviations or medians and interquartile ranges, as appropriate.

Exploratory factor analysis (EFA) was performed to identify latent constructs influencing burnout and work satisfaction. Factors were extracted using maximum likelihood estimation, with Promax rotation to allow for correlated factors. Internal consistency of the factors was assessed using Cronbach’s alpha.

To validate the latent constructs and their interrelationships, structural equation modeling (SEM) was applied. Model fit was evaluated using the Comparative Fit Index (CFI), Tucker-Lewis Index (TLI), and Root Mean Square Error of Approximation (RMSEA). Robust standard errors were used to account for non-normality, and standardized estimates were reported.

Participants were stratified into risk groups ("At Risk"and"Not at Risk"for burnout) using k-means cluster analysis based on factor scores derived from EFA. Associations between categorical variables were evaluated using chi-square tests, with Cramér’s V used to measure the strength of associations.

To identify predictors of burnout risk, logistic regression models were constructed, adjusting for demographic and professional characteristics. Results were reported as odds ratios (OR) with 95% confidence intervals (CIs). A multivariable logistic regression model was then fitted to evaluate the association of covariates with burnout risk. Statistical significance was defined as *p* < 0.05.

## Results

Data from 106 young Internists (age < 40 years) working in university- and non-teaching hospitals were collected. 56 (53.3%) respondents were males. The median age of participants was 30 years. A total of 89 (84%) were internal medicine residents, while 17 (16%) were specialists in Internal Medicine. Among the residents, 42 (46.7%) were in the first 2 years of their residency, 12 (13.3%) in the third year and 36 (40%) in their last 2 years of training. At the time of the survey, 83 respondents (78.3%) worked at university hospitals, while 23 (21.7%) were employed in non-teaching hospitals. Regarding the hospital size, 44 (41.9%) were working in hospitals with more than 1.000 beds, 34 (32.4%) in hospitals with a number of beds ranging from 500 to 1.000 beds, and 27 (25.7%) in hospitals with less than 500 beds (Table 1).

**Table 1:** General characteristics of respondents

Characteristics of respondents		
*Age (yr), median (range)*		30 (24–40)
< 30 yr, *n* (%)		50 (47.1)
30–34 yr, *n* (%)		38 (35.9)
35–40 yr, *n* (%)		18 (17)
*Gender*		
Male, *n* (%)		56 (53.3)
Female, *n* (%)		47 (44.7)
Resident physicians, *n* (%)		89 (84)
*Years of Residency*		
1°–2° year, *n* (%)		42 (46.7)
3° year, *n* (%)		12 (13.3)
4°–5° year, *n* (%)		36 (40)
Internal medicine specialists, *n* (%)		17 (16)
*Years since residency*		
1 year or less, *n* (%)		1 (5.9)
2–3 years, *n* (%)		6 (35.3)
4–5 years, *n* (%)		5 (29.4)
6 + years, *n* (%)		5 (29.4)
Physicians working at University Hospital, *n* (%)		83 (78.3)
*Hospital capacity*		
< 500 beds, *n* (%)		27 (25.7)
500–1000 beds, *n* (%)		34 (32.4)
> 1000 beds, *n* (%)		44 (41.9)
*Working hours*		
< 7 h, *n* (%)		22 (23.1)
7–9 h, *n* (%)		56 (58.9)
> 9 h, *n* (%)		17 (17.9)
*Type of patients managed*		
Inpatients, *n* (%)		15 (15.8)
Outpatients,* n* (%)		2 (2.1)
Both inpatients and outpatients, *n* (%)		78 (82.1)
Physicians at risk of burnout, *n* (%)		32 (37.2)

*n* number of respondents

**Fig. 1:**
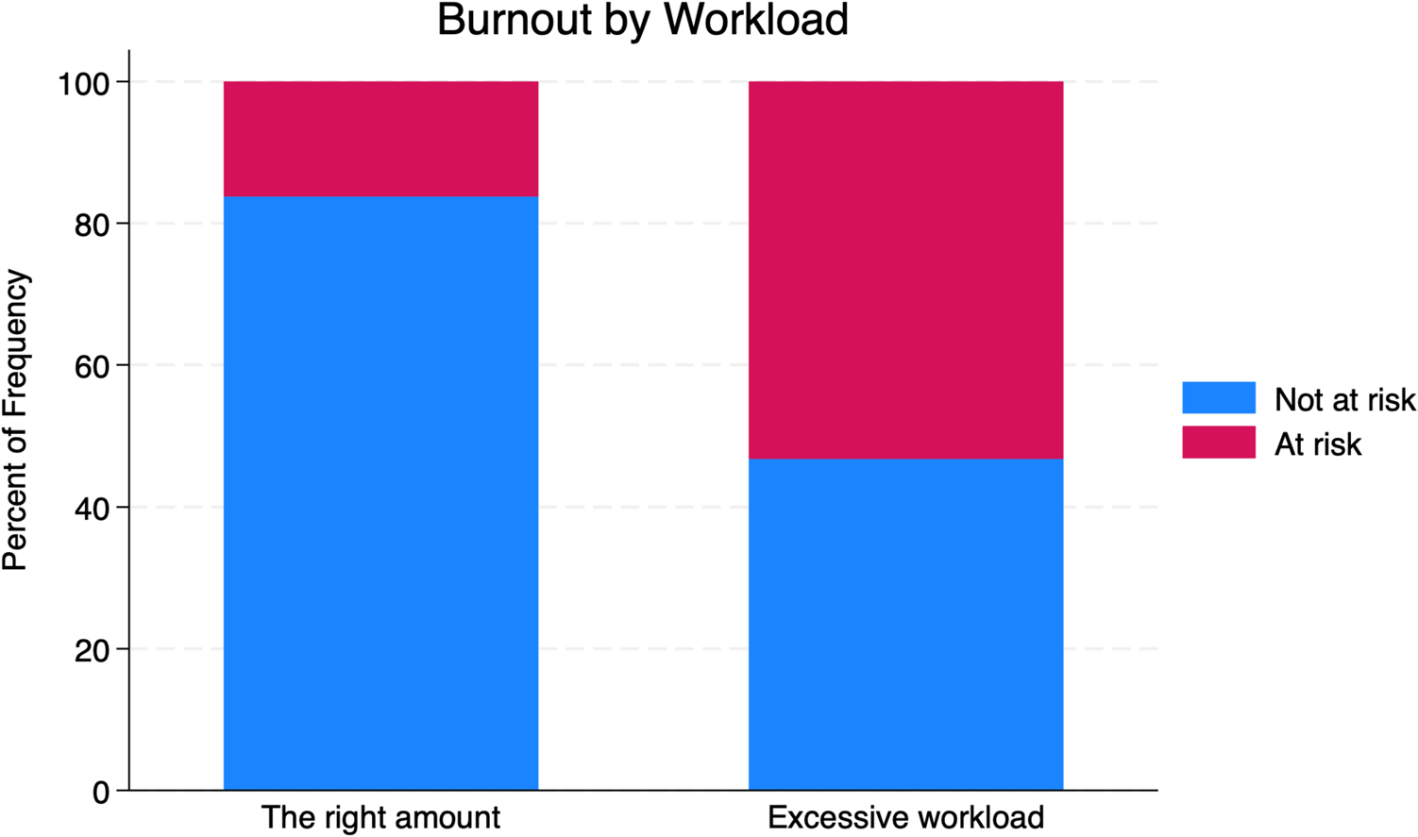
The bar chart depicts the percentage of individuals at risk (red) and not at risk (blue) of burnout based on workload. Two categories of workload are shown:"The right amount"and"Excessive workload."Percentages are calculated based on the total frequency within each workload category

**Fig. 2:**
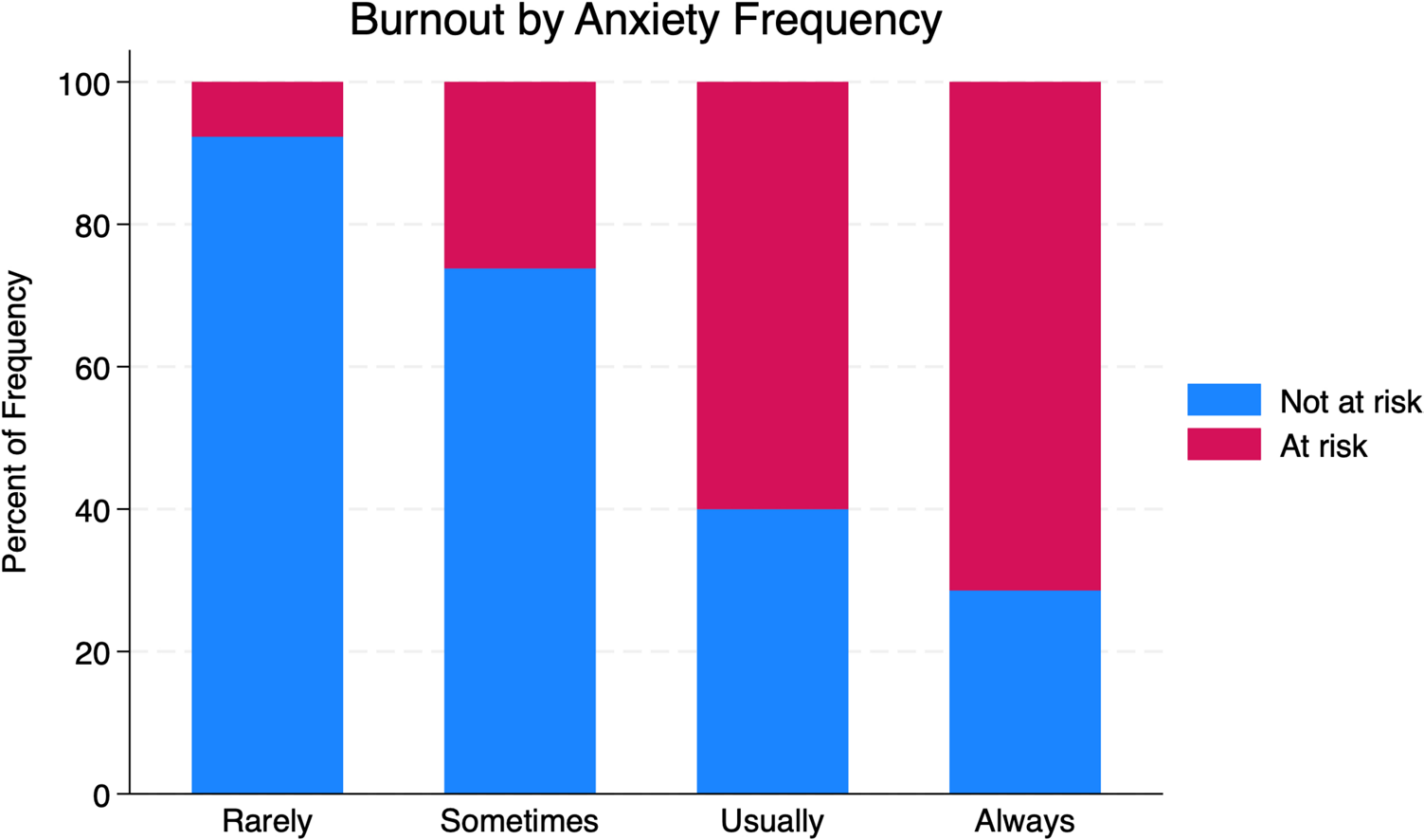
The bar chart shows the percentage of individuals at risk (red) and not at risk (blue) of burnout across four categories of anxiety frequency: rarely, Sometimes, Usually, and Always. Percentages were calculated based on the total frequency within each anxiety category

Burnout was reported by 37.2% of the respondents. The risk of burnout was associated with overall workload (Cramér’s V = 0.27; *p* = 0.001) (Fig. 1), satisfaction with the level of knowledge for the year of residency (Cramér’s V = 0.24; *p* = 0.004), and anxiety (Cramér’s V = 0.29; *p* = 0.002) (Fig. 2).

**Fig. 3:**
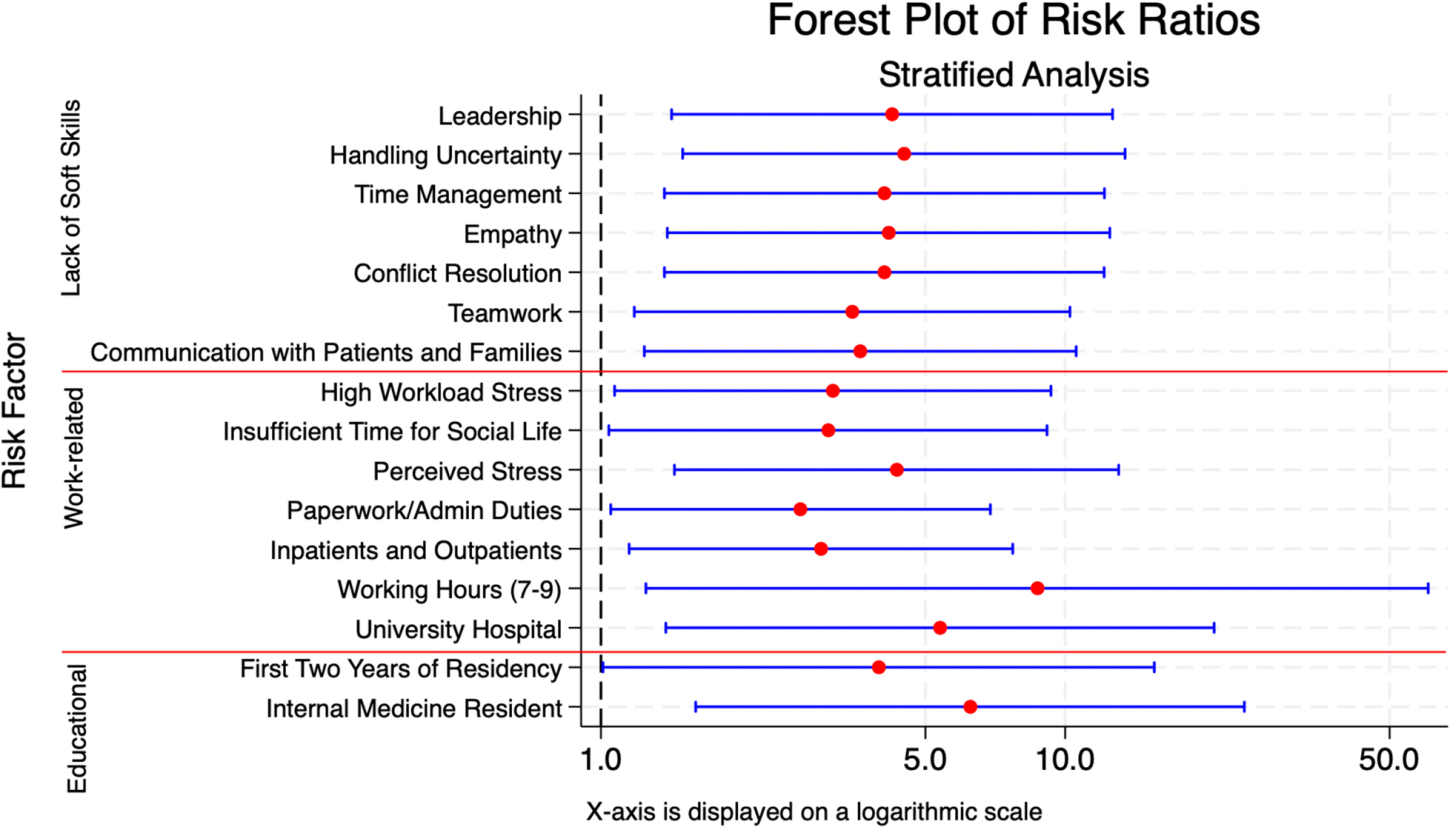
The forest plot displays risk ratios (red dots) with 95% confidence intervals (blue lines) for various risk factors grouped into three categories: lack of Soft Skills, Work-related, and Educational. The x-axis is shown on a logarithmic scale. The dashed vertical line at 1 represents the null hypothesis of no association. Factors with risk ratios and confidence intervals entirely to the right of the dashed line indicate a significant association with increased risk. Horizontal red lines separate the three categories for clarity

Also, burnout was also associated with being an Internal Medicine Resident (RR 6.25; 95% CI 1.60–24.32), especially in the first 2 years of residency (RR 3.97; 95% CI 1.01–15.56); working in an university hospital (RR 5.38; 95% CI 1.38–20.94), required working hours by the hospital ranging between 7 and 9 (RR 8.72; 95% CI 1,25–60.54), managing both inpatients and outpatients (RR 2.98; 95% CI 1.15–7.71), spending too much time on paperwork and administrative duties (RR 2.69; 95% CI 1.05–6.90), and presence of stress factors (RR 4.34; 95% CI 1.44–13.04) (Fig. 3). Among stress factors, burnout was associated with stress related to insufficient time for family, friends, and social interactions (RR 3.09; 95% CI 1.04–9.14) and stress related to high workload (RR 3.16; 95% CI 1.07–9.32) (Fig. 3).

Burnout was also associated with the absence of training or guidance on communication skills with patients and their family members (RR 3.62; 95% CI 1.24–10.56), teamwork (RR 3.48; 95% CI 1.18–10.24), conflict resolution (RR 4.08; 95% CI 1.37–12.13), empathy (RR 4.17, 95% CI 1.39–12.48), time management (RR 4.08; 95% CI 1.37–12.15), handling uncertainty (RR 4.50; 95% CI 1.50–13.46) and leadership (RR 4.24; 95% CI 1.42–12.65) (Fig. 3).

**Fig. 4:**
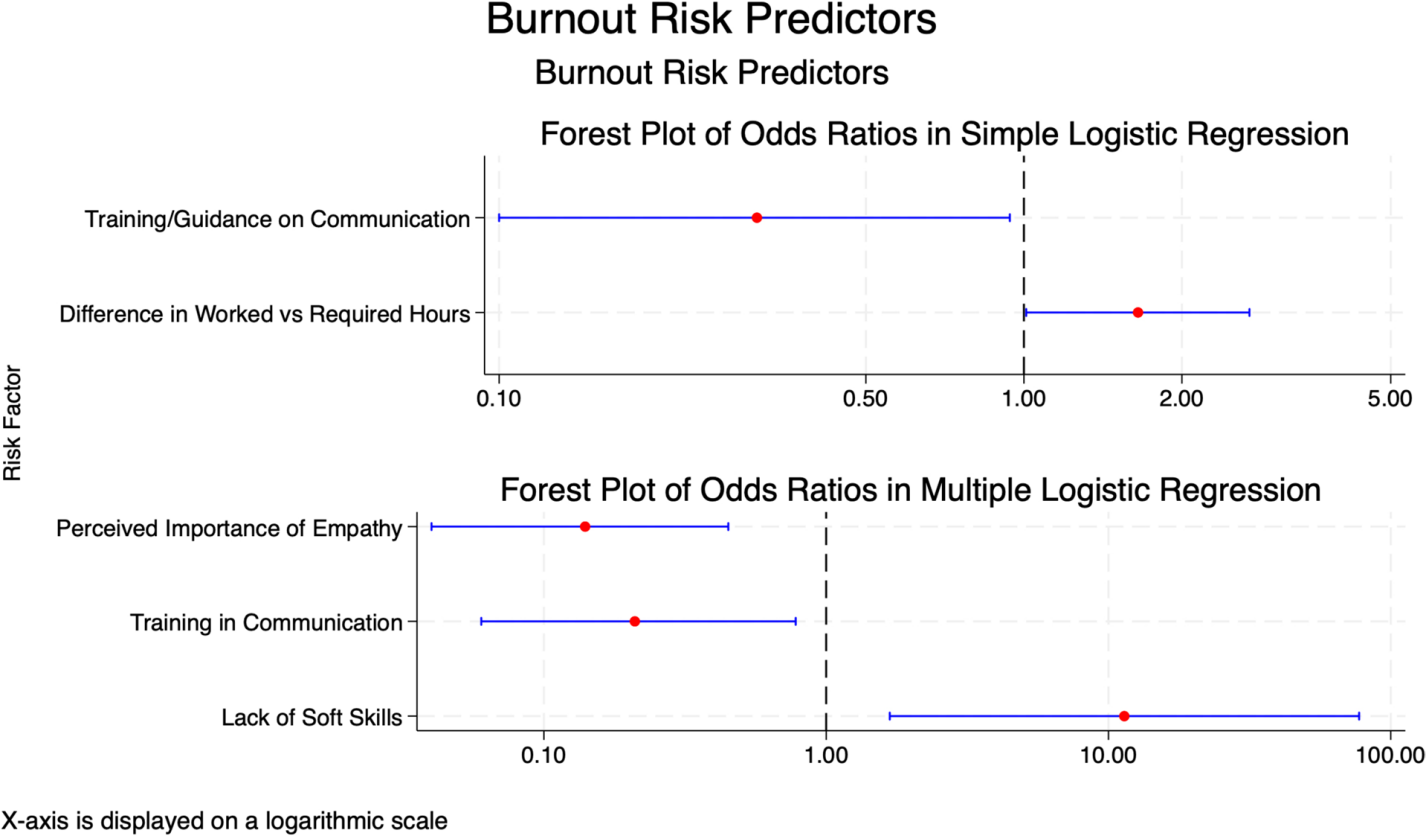
The top panel shows odds ratios with 95% confidence intervals for predictors of burnout based on simple logistic regression, including"Training/Guidance on Communication"and"Difference in Worked vs Required Hours."The bottom panel presents results from multiple logistic regression, including"Perceived Importance of Empathy,""Training in Communication,"and"Lack of Soft Skills."Red dots represent the odds ratios, blue lines indicate 95% confidence intervals, and the dashed vertical line at 1 denotes no effect. The x-axis is displayed on a logarithmic scale. Predictors with confidence intervals entirely to the right or left of the dashed line indicate significant associations

Logistic regression analysis showed a positive association between the risk of burnout and the difference between hours effectively worked and those required by the hospital (odds ratio, 1.65; 95% CI, 1.01 to 2.69; *p* = 0.045) (Fig. 4). The model included 51 observations, with a likelihood ratio chi-square of 4.50 (*p* = 0.034), a log-likelihood of − 30.21, and a pseudo-R^2^ of 0.0692. Burnout was negatively associated with training or guidance for communication skills with patients and their family members (odds ratio, 0.31; 95% CI, 0.10 to 0.94; *p* = 0.04) (Fig. 4). The model included 86 observations, with a likelihood ratio chi-square of 4.75 (*p* = 0.029), a log-likelihood of − 54.39, and a pseudo-R^2^ of 0.0419.

Moreover, logistic regression analysis showed a significant association between burnout risk and three factors: the lack of soft skills, training in communication, and the perceived importance of empathy. The lack of soft skills was associated with higher odds of burnout risk (odds ratio, 11.38; 95% CI, 1.68 to 77.26; *p* = 0.013) (Fig. 4). Communication training was inversely associated with burnout risk (odds ratio, 0.21; 95% CI, 0.06 to 0.78; *p* = 0.020) (Fig. 4). Similarly, the perceived importance of empathy was associated with a reduced likelihood of burnout (odds ratio, 0.14; 95% CI, 0.04 to 0.45; *p* = 0.001) (Fig. 4). The model included 86 observations, with a likelihood ratio chi-square of 26.08 (*p* < 0.001), a log-likelihood of − 43.72, and a pseudo-R^2^ of 0.2297.

Among all the respondents, 3 (2.8%) reported the presence of a support system to deal with burnout within their hospital and 16 (19%) of the respondents would not choose Internal Medicine Residency if they had the opportunity to choose their specialty again.

## Discussion

Several are the main findings of the present investigation. Firstly, burnout represents a common feature of young Italian Internal Medicine physicians, being detectable in more than one-third of the study population. Secondly, burnout was mainly associated with external factors, such as workload and working setting; in addition, the lack of guidance and training for soft skills represents an important factor associated with burnout.

Burnout has become a growing problem among healthcare professionals in the last decades. Rothenberger and colleagues, in their study enrolling physicians from 24 different specialties, showed an upward trend in the incidence of burnout from 2011 to 2014 in all specialties, especially in emergency medicine, urology, orthopedics, internal medicine, and anaesthesiology [10].

The increased prevalence could be explained by the older age, major complexity, and increased fragility of patients treated in Internal Medicine settings than in previous decades. The management of these patients is more challenging and demanding, thus increasing the stress and the feeling of exhaustion among doctors [11]. Moreover, discharging these patients has become very complex due to an inadequate domestic/social environment to support them thereafter [12, 13]. The fragility of internal medicine patients puts them at high risk of complications related to hospitalization, such as infections and bedsores, which in turn represent the main cause of arguing between family and doctors, increasing their level of stress and frustration, ending in burnout [14]. Finally, the increased amount of paperwork required from hospitals and administrative duties puts internists in a condition to use their work time in filling these documents instead of dedicating it to their patients [15].

In this study, the prevalence of burnout among young Italian internists was 37.2%. A recent study evaluating the prevalence of burnout among Internal Medicine specialists in Japan reported the prevalence of burnout pre-COVID and post-COVID as 34.6% and 34.5%, respectively, corroborating our findings. [16]. Another study, published in 2021, enrolling Spanish internists during the COVID-19 outbreak, reported a prevalence of 40.1% similar to our findings. [17] In their study, burnout was independently related to the assistance of patients with SARS-CoV-2, overworking without any compensation, and the fear of being contagious to their relatives [17].

In line with a recent report [18], being an internal medicine resident, especially in the first 2 years of residency, is associated with an increased risk of burnout. Notably, an insufficient learning environment can accelerate the levels of stress among resident doctors, who may feel inadequate and unable to cope with the increased responsibility [19]. In this study, the risk of burnout was also associated with the satisfaction for the level of knowledge and the related level of stress.

Burnout was higher among those working in university hospitals compared to their counterparts working in non-teaching hospitals. Working in university hospitals may seem to be less stressful because of continuous guidance, but the demands, expectations, workload, and complexity of cases seen may add up to the burnout state. This finding is also consistent with a previous study showing a high prevalence of burnout in academic hospitalists due to interference by medical students and having to balance the ability to perform teaching, research, and clinic with high demands from superiors and patients [20]. Physicians managing both inpatients and outpatients were found to have an increased risk of developing burnout. This can be a consequence of the excessive workload, long working hours, and the impairment of work–life balance as reported in previous studies [21, 22].

The present study showed that poor work–life balance and longer working hours were associated with burnout. Despite work-hour restrictions in Italy, many residents and tutors do not follow these restrictions, leaving limited time for wellness and spending time with family members/friends. Additionally, outside of the hospital, residents are often engaged in research work, answering emails, and studying, among others, thereby increasing their fatigue levels and impeding self-care [22].

The present investigation showed that training or guidance for soft skills was associated with a low risk of burnout. Previous research suggests a link between inadequate communication skills training and burnout [23]. One of the most frequently cited stressors is breaking bad news, particularly when physicians do not feel adequately trained in communication [24]. Moreover, conflicts within the healthcare team, especially if unresolved, might raise stress levels among physicians, leading to burnout [24].

Despite the high prevalence of burnout reported by the respondents in the present study, only 2.8% of them reported the presence of a support system to deal with burnout in their hospitals. Burnout, if unrecognized and untreated, leads to loss of productivity at work, dissatisfaction, and regrets about career choices [25]. Moreover, according to a study enrolling internists and cardiologists, although the sample size was small, burnout was strongly associated with medical errors [26].

### Future perspectives

Due to the high prevalence of burnout among young Italian Internists and its implications, measures to prevent or face burnout should be encouraged. Successful intervention for burnout should consider its multiple causes to help with a more holistic approach by applying stress-reducing techniques, having discussions on specific professional high-stress situations, and improving interpersonal skills training [27]. Moreover, organizational approaches involving programs focused on the work environment are necessary with the goal of reducing work overload.

Results of the present study also suggest the need for improving soft skills among young Italian internists. Effective communication skills and empathy with patients and their relatives help to reduce misunderstandings and improve doctor–patient relationships, resulting in reduced frustration [28]. Various approaches to enhance communication skills have been explored to address this issue [23]. Conflict resolution and teamwork provide avenues to handle disagreements constructively and create a less stressful and more enabling work environment [29]. Finally, leadership and time management help the team to prioritize tasks and set realistic goals, creating a better work-life balance. Investing in soft skills training during residency in Internal Medicine can equip future internists to handle stress, communicate better, and maintain a healthy work–life balance, preventing or delaying the onset of burnout.

Support system services, including employee assistance programs to face burnout, should be present in every hospital to manage physicians who experienced burnout from the budding stage with the aim of a quick recovery to avoid negative consequences. Additionally, this will help prevent the relapse of doctors who have already experienced burnout.

### Limitations

The present study has limitations: firstly, the small sample size and the study's design. Secondly, a sampling bias could be present in the study, as with all online surveys, that are completed only by persons who have access to the internet and social networks where it was shared and by those who are interested in the subject. Furthermore, it was not possible to extrapolate the distribution of the respondents in the country nor whether some of them work in the same hospital. Finally, the present survey mostly involves Internal Medicine Residents, with a low response rate from Specialists; further efforts are needed to include data from this category.

## Conclusion

Burnout is very common among young Italian Internists and is associated with work-related factors such as work overload; in addition, lack of guidance for soft skills represents another factor associated with burnout. These preliminary results need further studies involving all cadres of medical staff to help develop strategies to combat burnout in health institutions. Measures to prevent burnout should focus on reducing work overload and implementing training for transversal skills. Finally, a support system to deal with burnout should be present in every hospital. 

## Data Availability

All data are available, upon motivated request, from the corresponding author.
